# High‐sensitivity cardiac troponin T as a predictor of acute Total occlusion in patients with non‐ST‐segment elevation acute coronary syndrome

**DOI:** 10.1002/clc.23128

**Published:** 2018-12-21

**Authors:** Rocío Baro, Sohaib Haseeb, Santiago Ordoñez, Juan P. Costabel

**Affiliations:** ^1^ Cardiology Department Instituto Cardiovascular de Buenos Aires (ICBA) Buenos Aires Argentina; ^2^ Division of Cardiology Queen's University Kingston Canada

**Keywords:** acute total occlusion, high sensitivity troponin, non‐ST‐elevation acute coronary syndrome

## Abstract

**Background:**

A large percentage of patients with non‐ST‐segment acute coronary syndrome (NSTE‐ACS) present with acute total occlusion (TO) of some major epicardial vessel that does not generate electrocardiographic changes. Ongoing research into the methods of accurately predicting acute TO have not yielded great success.

**Hypothesis:**

High‐sensitivity cardiac troponin T (hs‐cTnT) has a good predictive value for the presence of acute TO of the culprit artery in patients with NSTE‐ACS.

**Methods:**

A single‐center retrospective study of 1011 patients diagnosed with NSTE‐ACS who underwent coronary angiography and hs‐cTnT measured on admission. The predictive value of hs‐cTnT in the presence of acute TO was assessed by the area under the ROC curve.

**Results:**

The mean age of the population was 67.12 ± 13.18 and 74.1% were male. 7.3% of the patients presented with acute TO. The AUC for hs‐cTnT to predict acute TO was 0.95. A hs‐cTnT value of 1006 ng/L (71.8 fold of the URL) best predicted the presence of acute TO, with a sensitivity of 86% and specificity of 95% positive predictive value (PPV): 86% and negative predictive value (NPV): 94%.

**Conclusions:**

Hs‐cTnT was a good predictor of acute TO in patients with NSTE‐ACS. Hs‐cTnT values greater than 1006 ng/L were highly predictive of acute TO of a major coronary vessel.

## 1 INTRODUCTION

Acute coronary syndromes (ACS) account for a large percentage of the cardiovascular disease burden and their early diagnosis and risk stratification is an emerging area of interest.^1^, ^2^, ^3^, ^4^ ACS refers to a spectrum of clinical presentations that include ST‐segment elevation myocardial infarction (STEMI), non‐ST‐segment elevation myocardial infarction (NSTEMI), and unstable angina. NSTEMI is currently the most frequent manifestation of ACS and represents the largest group of patients to undergo percutaneous coronary intervention (PCI). Despite advances in medical and interventional treatments, the morbidity and mortality of NSTEMI patients remains high.^5^ Early invasive strategy with PCI has shown better clinical and angiographic outcomes in high risk patients with NSTEMI than conservative management or late PCI.^6^ In this respect, there is a large percentage of patients with total occlusion (TO) of the culprit artery, diagnosed angiographically, presenting with NSTEMI.^7^, ^8^ Recent findings suggest that NSTEMI patients with TO of the culprit artery are at an increased risk of all‐cause mortality and major adverse cardiac events.^9^, ^10^ It is difficult to predict TO of the culprit artery in NSTEMI patients due to the lack of classic electrocardiograms (ECG) findings. Attempts have been made to find a marker that reliably predicts the presence of TO in patients with non‐ST‐segment acute coronary syndrome (NSTE‐ACS)—though without success. In this study, we evaluate the capacity of high‐sensitivity cardiac troponin T (hs‐cTnT) to predict acute TO of a coronary artery in patients presenting with NSTE‐ACS.

## 2 METHODS

### 2.1 Study design

Patients who were admitted with a final diagnosis of ACS were screened from the Buenos Aires Cardiovascular Institute between November 2013 and March 2017. The inclusion criteria were: (a) 18 years of age or older; (b) NSTE‐ACS; and (c) coronary angiography within 3 days of admission. Patients who presented with cardiac arrest or ST‐segment elevation acute coronary syndrome were excluded. All patients provided written informed consent for participation in the study. This study was approved by the local institutional research ethics committee, and conducted in accordance with the principles of the Declaration of Helsinki.

### 2.2 Study outcomes

The primary outcome was to assess the diagnostic accuracy of hs‐cTnT to predict acute TO in patients with NSTE‐ACS.

### 2.3 Diagnostic adjudication

The final diagnosis was adjudicated for all patients by independent cardiologists, with discrepancies resolved until a consensus was reached. NSTEMI was defined as the absence of ST‐segment elevation consistent with an infarction of ≥2 mm in contiguous chest leads, ST‐segment elevation with an infarction of ≥1 mm in two or more standard leads, or a new left bundle branch block, and the presence of positive cardiac necrosis markers.

### 2.4 Data collection and follow‐up

The baseline demographics, type of presentation, and hospital outcomes were recorded. All available clinic charts, (ECG, holter monitors, and cardiac imaging were reviewed. All the patients included in this study were followed‐up during hospitalization and evaluated for the presence of myocardial infarction, recurrent angina, stroke, and all‐cause mortality.

### 2.5 Blood sampling and laboratory methods

Venous blood samples were drawn upon arrival to the emergency department via a peripheral venous line and immediately processed. The Elecsys Troponin T‐high sensitive assay (Roche Diagnostics, Risch‐Rotkreuz, Switzerland) was used to measure hs‐cTnT concentrations with a limit of blank and limit of detection at 3 and 5 ng/L respectively, an imprecision corresponding to 10% coefficient of variation at 13 ng/L, and the 99th percentile upper reference limit from healthy individuals defined at 14 ng/L.^11^

### 2.6 Determination of coronary occlusion

The type of coronary artery lesion was determined according to the American College of Cardiology/American Heart Association (ACC/AHA) classification.^12^ The culprit vessel was identified based on the findings of coronary angiography, 12‐lead ECG, 2‐dimensional echocardiogram, and noninvasive stress test, as appropriate. All patients underwent PCI within 3 days of admission. Coronary flow pre and post‐PCI was classified according to the Thrombolysis in Myocardial Infarction (TIMI) risk score. Patients were divided into two groups (TO and Non‐TO group) based on the presence of pre‐TIMI flow; pre‐TIMI flow 0 (TO group), and pre‐TIMI flow ≥1 (non‐TO group). The differentiation between acute and chronic TO was based on the following factors: morphology of the occlusion (presence of a fresh thrombus, bridge, and ipsi‐or‐contralateral collaterals), the ECG recording, echocardiographic findings, and prior documented acute coronary events in the same territory.

### 2.7 Statistical analysis

Data were collected in an Excel (Microsoft, Redmond, WA) file and imported into IBM SPSS (version 20.0 for Macintosh, Chicago, IL) for statistical analysis. Continuous variables were described as mean (SD) or median (interquartile range, IQR) as appropriate. Categorical variables were described as frequencies and percentages. Independent sample *t* tests were used to compare the normally distributed continuous variables, the Mann‐Whitney *U* was used for non‐normally distributed continuous variables, and the Pearson chi‐squared test (or the Fisher's Exact test as applicable) for categorical variables. The area under the receiver operating characteristic (ROC) curve (AUC) was used to quantify the diagnostic accuracy of the study endpoint. Univariate and multivariate logistic regressions were used to assess the predictive accuracy of hs‐cTnT to predict acute TO. A two‐sided *P*‐value of less than 0.05 was considered statistically significant.

## 3 RESULTS

A total of 1011 patients were identified who met the inclusion/exclusion criteria. The mean age was 67.12 SD 13.18 years, 74.1% were male, 26.7% had a history of coronary artery disease, and 8.9% had undergone coronary artery bypass graft surgery (Table 1). The adjudicated diagnosis was NSTEMI in 608 (60.1%) patients and unstable angina in 403 (39.8%) patients.

**Table 1:** Baseline demographics of the population

Variable	All patients (n = 1011)	TO (n = 74)	Non‐TO (n = 937)	*P*‐value
Age, years	67.12 ± 13.18	67.00 ± 15	66.00 ± 14	0.235
Male, n (%)	749 (74.1)	52 (70.2)	697 (74.4)	0.496
Current smoker, n (%)	169 (16.7)	38 (51.3)	131 (14.0)	0.001
Prior smoker, n (%)	400 (39.6)	48 (64.0)	352 (37.5)	0.001
Hypertension, n (%)	740 (73.2)	66 (89.0)	674 (72.0)	0.001
Dyslipidemia, n (%)	597 (59.1)	54 (46.5)	553 (59.0)	0.019
Diabetes mellitus, n (%)	233 (23.1)	17 (23.0)	216 (23.0)	1.000
Peripheral vascular disease, n (%)	136 (13.5)	9 (13.0)	131 (13.5)	0.121
Renal failure (ClCr < 60), n (%)	72 (7.1)	10 (13.5)	62 (6.6)	0.034

Abbreviation: TO, total occlusion.

The median Global Registry of Acute Coronary Events score (GRACE) risk score of the population was 118 (IQR: 105‐131) and the median TIMI risk score was 3 (range: 2‐4). The median GRACE risk score was significantly higher in the TO group of patients (131 vs 117; *P* = 0.032) (Table 2 **)**. The median hospital stay was 4 (IQR: 3‐5.3) days. In‐hospital adverse events included recurrent angina (4.5%), myocardial reinfarction (3.5%), stroke (0.4%), bleeding (6.3%), arrhythmias (2.0%), and acute kidney failure (4.4%). In‐hospital adverse events stratified by patients with vs without acute TO are summarized in Table 3. During hospitalization, 8% of the patients required myocardial revascularization surgery.

**Table 2:** Values of the TIMI and GRACE risk scores stratified by patients with vs without acute TO

Variable	Median (IQR)	TO (n = 74)	Non‐TO (n = 937)	*P*‐value
TIMI score	3 (2–4)	3 (3‐4)	3 (2‐4)	0.854
GRACE score	118 (105–131)	131 (120‐140)	117 (104‐126)	0.032

Abbreviations: IQR, interquartile range; TIMI, Thrombolysis in Myocardial Infarction; TO, total occlusion.

**Table 3:** In‐hospital adverse events stratified by patients with vs without acute TO

Variable	All patients (n = 1011)	TO (n = 74)	Non‐TO (n = 937)	*P*‐value
Recurrent angina, n (%)	46 (4.5)	10 (13.5)	36 (0.3)	0.001
Re‐infarction, n (%)	35 (3.5)	7 (9.4)	28 (0.3)	0.010
Stroke, n (%)	4 (0.4)	1 (1.3)	3 (0.3)	0.262
Mortality, n (%)	14 (1.3)	6 (8.0)	8 (0.8)	0.001
Bleeding, n (%)	63 (6.3)	5 (6.7)	58 (0.6)	0.802
Acute kidney failure, n (%)	45 (4.4)	8 (10.8)	37 (0.4)	0.013
Arrhythmias, n (%)	20 (2.0)	10 (13.5)	10 (0.1)	0.001

Abbreviation: TO, total occlusion.

Angiographic parameters are summarized in Table 4 *additional information*. Coronary angiography identified 74 (7.3%) patients with acute TO. Of these, 40 (54%) patients had TO of the circumflex artery or its main branches, 25 (34%) patients had TO of the right coronary artery, and 9 (12%) patients had TO of the anterior descending artery. The total mortality was 1.3% (6 patients in TO‐group and 8 patients in non‐TO group).

**Table 4:** Angiographic parameters

Variable	All patients (n = 1011)	TO (n = 74)	Non‐TO (n = 937)	*P*‐value
Drug eluting stent implanted, n (%)	970 (96%)	72 (97%)	898 (96%)	0.762
Length of stent implanted, mm	3.7 (2.3‐4.2)	3.2 (2.1‐3.6)	3.6 (2.3–4.2)	0.041
Number of vessels treated, n	2 (1–3)	1(1–2)	2(1‐3)	0.070

Abbreviation: TO, total occlusion.

**Figure 1:**
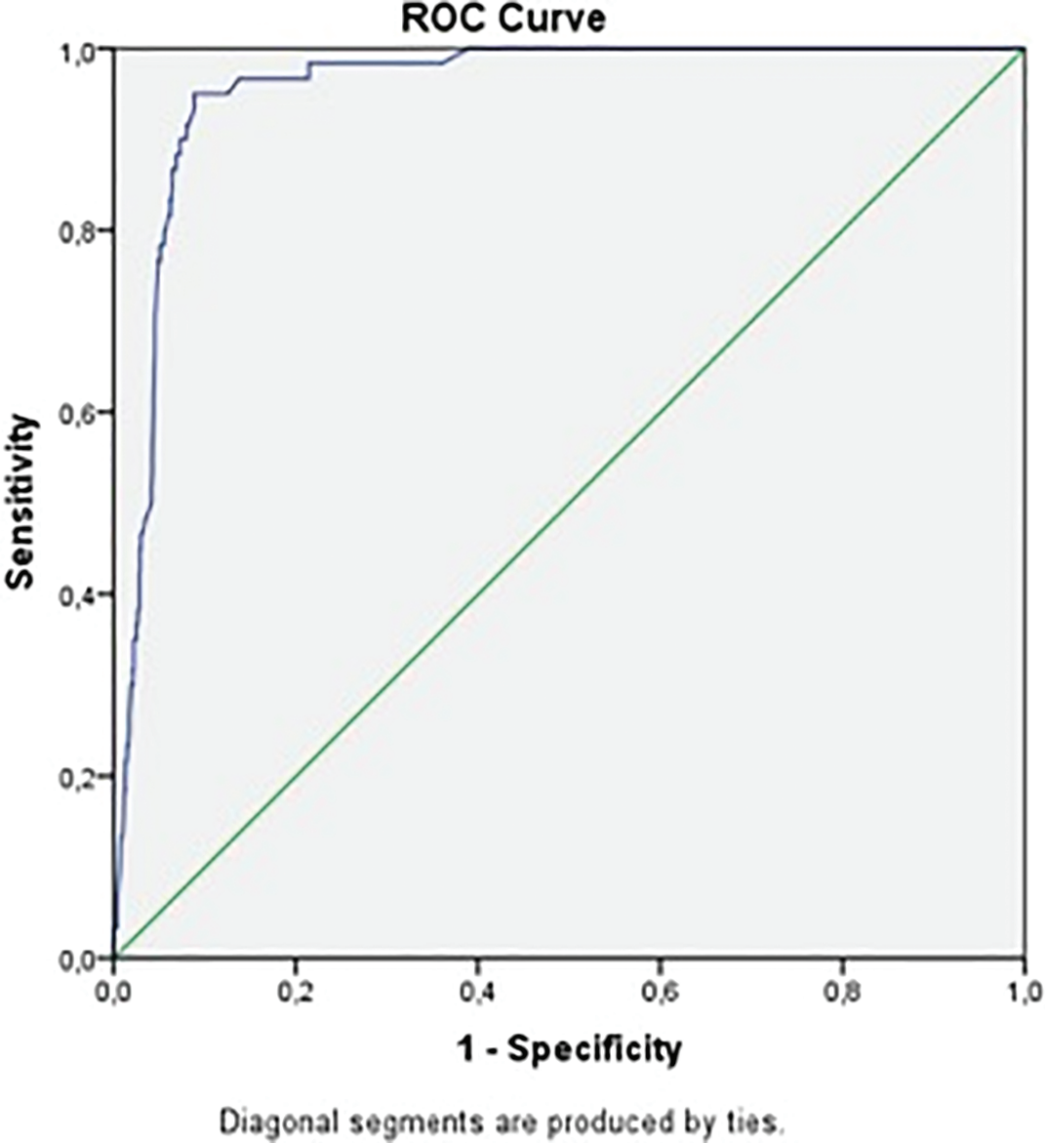
Receiver operating characteristic (ROC) curve for high‐sensitivity cardiac troponin T (hs‐cTnT) and acute total occlusion (TO)

The global median hs‐cTnT concentration on admission was 17 ng/L (1.2‐fold of the URL) (IQR: 11‐78.25). The median hs‐cTnT of patients with acute TO was 1473 ng/L (105.2‐fold of the URL) (IQR: 1234‐1977) while, the median hs‐cTnT without acute TO was 15 ng/L (1.1‐fold of the URL) (IQR 9‐22) (*P* < 0.001). Hs‐cTnT concentrations were an independent predictor of acute TO on multivariate analysis (*P* < 0.001). The prognostic accuracy of hs‐cTnT to predict acute TO—as quantified by the area under the ROC curve—in patients with NSTE‐ACS was 0.95 (95% confidence interval 0.93‐0.97) (Figure 1). A hs‐cTnT value of 1006 ng/L (71.8‐fold of the URL) best predicted the presence of acute TO, with a sensitivity of 86% and specificity of 95% (PPV: 86% and NPV: 94%).

## 4 DISCUSSION

In a retrospective cohort study of patients with NSTE‐ACS, we have demonstrated that hs‐cTnT can be a useful marker of predicting the presence of acute TO. We report a number of clinically relevant findings. First, hs‐cTnT concentrations were significantly higher in patients with acute TO and that hs‐cTnT was an independent predictor of acute TO. Second, a cutoff value of 1006 ng/L hs‐cTnT showed good accuracy for the diagnosis of acute TO.

The prevalence of acute coronary occlusions in NSTEMI patients varies from 19% to 30%.^13^ In our study, the prevalence of acute occlusions was 7.3%, which could be due to the fact that our population was at a lower risk and included unstable angina patients than the other published series. In Khan et al's meta‐analysis to estimate the difference in outcomes between TO and non‐TO patients with NSTEMI,^9^ the right coronary artery was occluded in most cases (40%) followed by the circumflex artery (33%). In our study, the circumflex artery was the most commonly involved vessel.

Multiple studies have reported short and long‐term adverse events of acute TO in patients with NSTEMI.^14^, ^15^, ^16^ However, there is little research regarding the complementary diagnostic methods to predict acute TO. Aijaz et al^6^ identified advanced age (57.6 ± 11.2 vs 60.0 ± 10.0; *P* = 0.03) and left ventricular ejection fraction (43.9 ± 12.2 vs 50.1 ± 10.1; *P* < 0.001) as predictors of an occluded coronary artery, however there was no significant differences observed in major adverse cardiovascular events in occluded vs non‐occluded arteries' patients. On the other hand, the occlusion of an epicardial artery leads to an alteration of cardiac parietal motility that can be evidenced by echocardiography.^17^

New echocardiographic techniques have been developed in the last decade to increase the prognostic accuracy of identifying acute TO. Eek et al,^17^ in a clinical trial, evaluated the ability of cardiac strain assessment by echocardiography to predict the presence of TO in patients with NSTE‐ACS. The authors demonstrated that the presence of four or more dysfunctional areas with a value of strain >14% was an independent predictor of TO in patients with NSTEMI, with a sensitivity of 85% and specificity of 70%. Additionally, the authors reported that the value of fourth generation cTnT was significantly higher in patients with TO (0.33 mg/L), which was able to identify these patients with a sensitivity of 77% and specificity of 78%. In our study, the echocardiographic evaluation was performed between 1 and 3 days after admission, therefore it would not be useful for early prediction of acute occlusions. It is also worth noting the echocardiographic method cannot identify alterations in segmental and global motility in patients who already present this type of alterations, especially in those with previous infarctions.

## 5 LIMITATIONS

This is a single‐center retrospective cohort study, which carries a potential for inherent bias. Although a large tertiary care center, a larger sample size with cross‐center comparisons is needed to further validate the results. Patients with NSTEMI were included based on documented coronary angiography at the indexed visit. It is possible that patients who did not undergo angiography due to a lower risk, presence of comorbidities, or clinical status were underrepresented in this analysis.

## 6 CONCLUSION

Hs‐cTnT was found to be a good predictor of acute TO in patients with NSTE‐ACS. A value of 1006 ng/L best predicted the presence of acute TO, with a sensitivity of 86%, and a specificity of 95%.

## CONFLICTS OF INTEREST

The authors declare no potential conflict of interests.
